# Biological Society of Philadelphia

**Published:** 1858-01

**Authors:** 


BIOLOGICAL SOCIETY OF PHILADELPHIA.—It gives us great pleasure to be enabled to announce the formation of a society in this city with the above title. Though we have no lack of medical societies in the United States, we are not aware that any exists having for its object the cultivation of biological science in its most enlarged sense.

The scope of action of the Biological Society of Philadelphia will be similar to that of the *Société de Biologie* of Paris. It will be pre-eminently a working society, and will endeavor, by encouraging original investigations, to enlarge our knowledge of the laws and conditions of life. The subjects which it is especially intended to cultivate are Physiology, Pathology, Microscopy, and Organic Chemistry. The society already numbers some of our most able and energetic men among its members, and will, we doubt not, succeed in the objects which have led to its formation.

In our next number we will announce the names of the officers.

---

WE have received from Dr. N. D. Benedict, of Magnolia, East Florida, a circular letter descriptive of the winter retreat for invalids, which he has had in operation at that place during the last two seasons.

Magnolia is upon the St. John's River, twenty-five miles south of Jacksonville, and readily accessible three times a week by comfortable steamers, in about thirty hours from Savannah. The house opened by Dr. Benedict is large and comfortable, having been expressly designed as a *maison de santé;* and the climate of that part of Florida is admirably adapted for those the state of whose health—whether from threatening of pulmonary disease or other affections —makes it desirable that they should avoid the rigors of a Northern winter.

